# TRPV1 antagonist attenuates postoperative hypersensitivity by central and peripheral mechanisms

**DOI:** 10.1186/1744-8069-10-67

**Published:** 2014-11-17

**Authors:** Eva Uchytilova, Diana Spicarova, Jiri Palecek

**Affiliations:** Department of Functional Morphology, Institute of Physiology, Academy of Sciences of the Czech Republic, Videnska 1083, 142 20 Prague, Czech Republic

**Keywords:** SB366791, TRPV1, Allodynia, Hyperalgesia, Surgical pain, Spinal cord

## Abstract

**Background:**

Acute postoperative pain is one of the frequent reasons for pain treatment. However, the exact mechanisms of its development are still not completely clear. Transient receptor potential vanilloid 1 (TRPV1) receptors are involved in nociceptive signaling in various hypersensitive states. Here we have investigated the contribution of TRPV1 receptors expressed on cutaneous peripheral nociceptive fibers and in the spinal cord on the development and maintenance of hypersensitivity to thermal and mechanical stimuli following surgical incision. A rat plantar incision model was used to test paw withdrawal responses to thermal and mechanical stimuli. The effect of the TRPV1 receptor antagonist SB366791 was investigated 1) by intrathecal injection 15 min before incision and 2) intradermal injection before (30 min) and immediately after the surgery. Vehicle-injected rats and naïve animals treated identically were used as controls.

**Results:**

Plantar incision induced mechanical allodynia and hyperalgesia and thermal hyperalgesia. A single intrathecal administration of SB366791 significantly reduced postincisional thermal hyperalgesia and also attenuated mechanical allodynia, while mechanical hyperalgesia remained unaffected. Local intradermal SB366791 treatment reduced thermal hyperalgesia and mechanical allodynia without affecting mechanical hyperalgesia.

**Conclusions:**

Our experiments suggest that both peripheral and spinal cord TRPV1 receptors are involved in increased cutaneous sensitivity following surgical incision. The analgesic effect of the TRPV1 receptor antagonist was especially evident in the reduction of thermal hyperalgesia. The activation of TRPV1 receptors represents an important mechanism in the development of postoperative hypersensitivity.

## Background

Postsurgical wound pain is primarily characterized by mechanical allodynia and thermal hyperalgesia. It is well established that hypersensitivity at the site of tissue injury is predominantly caused by the sensitization of primary afferent nerve fibers, of which the vast majority are nociceptors [1], and by the modulation of nociceptive transmission at the spinal cord level [2, 3] where enhanced AMPA receptor function plays a significant role [4, 5]. Using a rat model of plantar incision [6], numerous studies have described the changes and mechanisms ongoing in the periphery and in the spinal cord that contribute to cutaneous hypersensitivity induced by surgery. Sensitization of a broad spectrum of sensory nerve fibers (C, Aδ and Aβ) occurs after incision at the peripheral and/or spinal cord level [4, 7]. In the peripheral tissues, neutrophils [8], acidosis [9], nerve growth factor (NGF) [10, 11], EP_1_ receptor [12], B_1_ and B_2_ bradykinin receptors, P2X purinoceptors, transient receptor potential vanilloid 1 (TRPV1) receptor, nitric oxide (NO) synthase, lipoxygenase [13], insulin-like growth factor 1 (IGF-1) [14], acid-sensing ion channel 3 [15], the activation of AMP-activated protein kinase that inhibits IL-6 mediated signaling to ERK [16] and others could contribute to postoperative pain. In the spinal cord cyclooxygenase (COX) [17, 18], the EP_1_ receptor for a product of COX metabolism - prostaglandin E_2_ [19], brain derived neurotrophic factor (BDNF) [20], glial cell activation and interleukin-1 beta (IL-1β) [21], the chemokine CCL2 [22], serotonin receptors [23], GABA_A_ and GABA_B_ receptors [24], calcium/calmodulin-dependent protein kinase IIα [5], p38 mitogen-activated protein kinase (p38 MAPK) [25], phosphatidylinositol 3-kinase (PI3K) [26] and others could contribute to the hypersensitivity induced by plantar incision.

The TRPV1 receptor is a ligand gated, non-selective cation channel abundantly expressed on the peripheral and central terminals of small diameter, primary afferent neurons [27]. Using TRPV1 knockout mice, it was shown that TRPV1 receptors are necessary for thermal hyperalgesia [28] and that they mediate the spontaneous firing and heat sensitization of cutaneous primary afferents after plantar incision [29]. The exogenous TRPV1 receptor agonist capsaicin was shown to produce a rapid degeneration of intracutaneous nerve fibers in humans after a single intradermal injection [30]. Local treatment with high concentration TRPV1 receptor agonists [30, 31] brings a highly selective regional analgesia by causing the degeneration of capsaicin-sensitive nociceptive fibers, resulting in significant and long lasting increases in nociceptive thresholds. In our previous work we have used a high concentration capsaicin intradermal injection (1.5 mg/0.1 ml) in the model of hindpaw incision to attenuate mechanical allodynia and hyperalgesia and to completely prevent thermal hyperalgesia [32]. Subcutaneous infiltration of a lower capsaicin concentration (100 μg/200 μl) also impaired the transduction of heat and chemical stimuli after plantar incision, while mechanical hypersensitivity was not affected [33]. In clinical trials, the analgesic effect of a single intraoperative wound instillation of capsaicin after open mesh groin hernia repair was shown in patients [34] and is being further tested in postoperative pain settings [35].

Numerous TRPV1 receptor antagonists have been developed and also tested in clinical trials [36]. In this study we have used SB366791, a potent and selective TRPV1 antagonist, with a good selectivity profile defined in a wide range of assays [37]. The aim of this study was to prevent TRPV1 receptor activation with antagonist treatment in order to further investigate the role of TRPV1 receptors in a model of postincisional pain. The main emphasis was placed on elucidating the role of peripheral and central TRPV1 receptors in the development and maintenance of postincisional hypersensitivity to mechanical and heat stimuli. We hypothesized that both peripheral and central TRPV1 receptors may play a significant role and that the specific blockade of TRPV1 receptors by local or intrathecal treatment with the antagonist could prevent the development of postoperative thermal hyperalgesia and hypersensitivity to mechanical stimuli.

## Results

### Intrathecal and intradermal TRPV1 receptor antagonist SB366791 treatment attenuated postincisional thermal hyperalgesia

**Figure 1:**
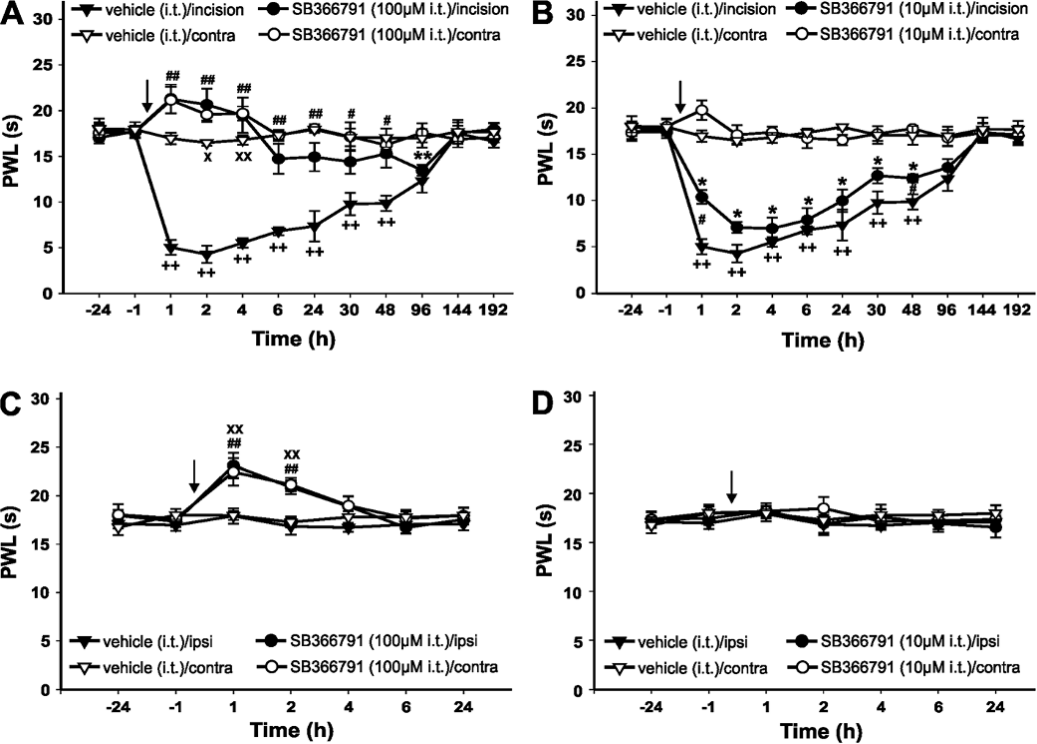
**Intrathecal injection of TRPV1 receptor antagonist attenuated thermal hyperalgesia induced by plantar incision. (A)** Vehicle injection 15 min before the surgery (time 0) did not affect the development of thermal hyperalgesia in the incised hindpaw and did not affect the PWL of the contralateral intact hindpaw (n = 6). Intrathecal injection of 100 μM SB366791 prevented the development of thermal hyperalgesia in the operated hindpaw (application 15 min before the surgery) and transiently increased PWL in both hindpaws (n = 6). **(B)** Intrathecal injection of 10 μM SB366791 (15 min before the surgery, n = 4) showed a moderate analgesic effect on postoperative thermal hyperalgesia when compared to the intrathecal injection of vehicle (n = 6). **(C)** Intrathecal injection of vehicle (15 min before sham surgery) did not result in any change of the mean PWLs in either hindpaw (n = 6). Intrathecal injection of 100 μM SB366791 (15 min before sham surgery) induced transient thermal hyposensitivity in both hindpaws (n = 6). **(D)** Intrathecal injection of vehicle (n = 6) or 10 μM SB366791 (n = 4, 15 min before sham surgery) did not change the PWL in comparison to the baseline values. *P <0.05, SB366791 injection: ipsilateral versus contralateral side; ^++^P <0.01, vehicle injection: ipsilateral versus contralateral side; ^#^P <0.05, ^##^P <0.01, SB366791 versus vehicle injection on the ipsilateral hindpaw; ^X^P <0.05, ^XX^P <0.01 SB366791 versus vehicle injection on the contralateral hindpaw. The time of drug administration is marked by arrow.

To examine the postincisional changes of the rat’s reactivity to heat, paw withdrawal latencies (PWLs) to thermal stimulation were tested on the plantar skin adjacent to the incision. The thermal sensitivity of the intact skin before the surgery did not differ between the hindpaws. Plantar incision caused a rapid decrease of the PWLs to thermal stimuli in animals in which the vehicle was applied intrathecally (Figure 1A,B). The mean withdrawal latency of about 17 s for the intact skin decreased to 5 s at one hour after the incision. The mean PWL reached its minimum during the first 4 hours following surgery, then it started to gradually return to the preincision values. At 6 days after the surgery, the sensitivity to heat of the incised hindpaw was not significantly different from its preincision values. These results suggest the presence of thermal hyperalgesia lasting several days after the plantar incision.

First, the role of spinal TRPV1 receptors in the development of the postincisional thermal hyperalgesia was investigated. Changes of PWLs to thermal stimuli after the intrathecal application of 100 μM SB366791 applied 15 min before the incision were examined (Figure 1A, circle symbols). Initially, a complete reversal of the postoperative thermal hyperalgesia was observed in SB366791-treated animals. The mean withdrawal latencies of the incised hindpaws were longer during the first 4 h after the surgery when compared to the baseline values. However, subsequently the PWLs started to gradually decrease, and at 96 h after the surgical procedure they were transiently significantly shorter when compared to the baseline. The PWLs to thermal stimuli returned to the preincision values 6 days after the surgery. Intrathecal injection of SB366791 also caused a significant increase of the mean withdrawal latencies of the contralateral intact hindpaws in these animals. PWLs remained prolonged for at least 4 h. Intrathecal pretreatment with TRPV1 receptor antagonist (100 μM) dramatically attenuated the thermal hyperalgesia induced by the plantar incision, while moderate transient thermal hypoalgesia was present for a few hours after the surgery.

In another series of experiments, the effect of intrathecal SB366791 application at a lower concentration (10 μM) was tested under the same conditions (Figure 1B, circle symbols). The 10 μM SB366791 pretreatment resulted in only a minor reduction of the thermal hyperalgesia present after the incision, when compared to the vehicle-treated animals. However, the difference between the SB366791- and the vehicle-treated groups reached statistical significance at 1 h and 48 h after the surgical procedure. On the sixth day the thermal sensitivity returned to its preincision values. Intrathecal pretreatment with the lower TRPV1 receptor antagonist concentration (10 μM) exhibited only a limited antinociceptive effect on incisional thermal hyperalgesia.

In the next series of experiments naïve animals were used, and changes of the PWLs to thermal stimuli after the intrathecal application of 100 μM SB366791 15 min before sham surgery (Figure 1C, circle symbols) or 10 μM SB366791 15 min before sham surgery (Figure 1D, circle symbols) were tested. The mean withdrawal latencies of both hindpaws increased after 100 μM TRPV1 antagonist treatment and remained prolonged for 2 h after sham surgery, suggesting that transient hyposensitivity to heat developed after intrathecal 100 μM SB366791 treatment. After 2 h the latencies of both hindpaws gradually returned to baseline. Intrathecal application of 10 μM SB366791 did not cause any significant changes of the PWLs of the intact hindpaws after sham surgery compared to baseline values. The thermal sensitivity of the intact plantar skin was not affected after the intrathecal application of 10 μM SB366791, while 100 μM concentration induced transient hypoalgesia lasting for a few hours in naïve rats.

**Figure 2:**
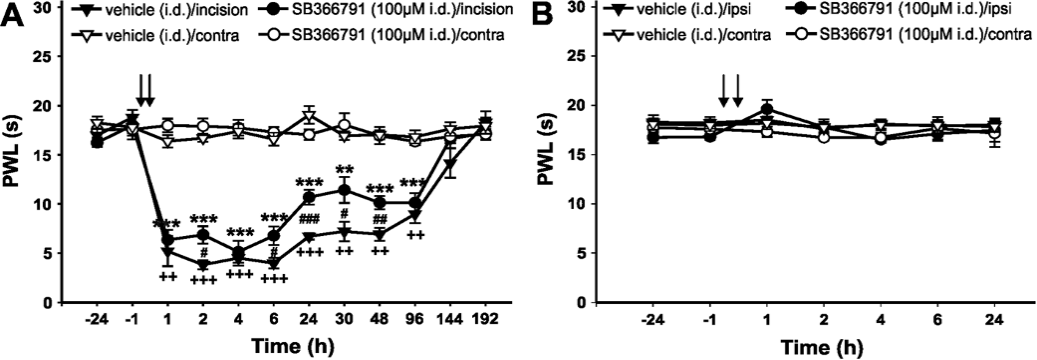
**Intradermal injection of SB366791 partially attenuated thermal hyperalgesia induced by plantar incision. (A)** Intradermal injection of vehicle 30 min before and immediately after the plantar incision did not affect thermal hyperalgesia measured as a significant decrease of PWL in the incised hind paw (n = 6). Intradermal injection of 100 μM SB366791 (30 min before and immediately after the surgery) had a significant analgesic effect on the postoperative thermal hyperalgesia (n = 8). **(B)** Intradermal injection of vehicle (n = 6) or 100 μM SB366791 (n = 4) 30 min before and immediately after the sham surgery did not affect the basal thermal sensitivity. **P <0.01, ***P <0.001 SB366791 injection: ipsilateral versus contralateral side; ^++^P <0.01, ^+++^P <0.001 vehicle injection: ipsilateral versus contralateral side; ^#^P <0.05, ^##^P <0.01, ^###^P <0.001 SB366791 versus vehicle injection on the ipsilateral hind paw. The time of drug administration is marked by arrow.

The role of peripheral TRPV1 receptors was tested by measuring changes of the PWLs to thermal stimuli after intradermal SB366791 treatment 30 minutes before and immediately after the incision (Figure 2A, circle symbols). The 100 μM SB366791 treatment did not prevent the PWL decrease after the incision. However, the TRPV1 antagonist significantly attenuated the development of postoperative thermal hyperalgesia in comparison to vehicle-treated incised hindpaws at some time points during the first two days after the surgical procedure. On the sixth day after the surgery, no difference in heat sensitivity in the operated and intact hindpaws was observed. Our control experiments in naïve animals showed that 100 μM SB366791 did not cause any changes in the thermal sensitivity of the intact plantar skin when applied intradermally 30 minutes before and immediately after sham surgery (Figure 2B). The analgesic effect of local SB366791 pre- and post-treatment on postoperative thermal hyperalgesia was less effective than the analgesia induced by intrathecal SB366791 pretreatment.

### The effect of intrathecal 100 μM SB366791 treatment on postincisional mechanical allodynia and hyperalgesia

**Figure 3:**
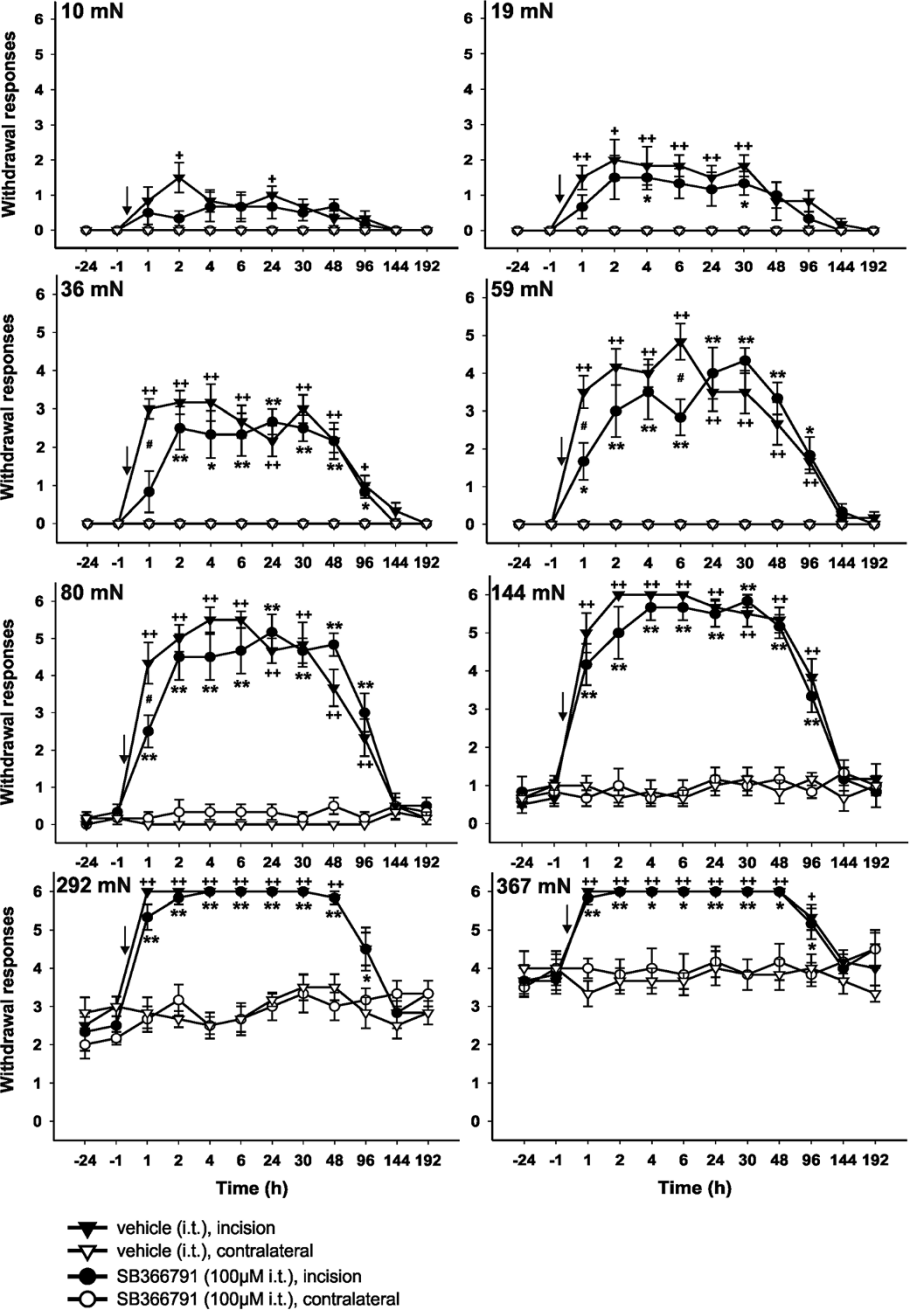
**The effect of intrathecal 100 μM SB366791 treatment on the responsiveness to mechanical stimuli after plantar incision.** The number of responses to a range of von Frey filaments applied to the plantar skin of both the incised and the contralateral intact hindpaws, before and after the surgical procedure, was measured with the same pattern and testing frequency. Testing the incised skin after the intrathecal injection of vehicle (15 min before the surgery) and the corresponding contralateral intact hindpaw (n = 6) indicated the development of postoperative mechanical allodynia (10, 19, 36, 59, 80 mN) and hyperalgesia (144, 292, 367 mN). Intrathecal injection of 100 μM SB366791 (15 min before the surgery) showed a moderate antinociceptive effect of the TRPV1 receptor antagonist on postoperative mechanical allodynia, while mechanical hyperalgesia remained unaffected (n = 6). *P <0.05, **P <0.01, SB366791 injection: incision versus contralateral side; ^+^P <0.05, ^++^P <0.01, vehicle injection: incision versus contralateral side; ^#^P <0.05, SB366791 versus vehicle injection on the incised hind paw. The time of drug administration is mark by arrow.

The role of spinal TRPV1 receptors was investigated with 100 μM SB366791 intrathecal application 15 min before the surgical incision. The changes in the reactivity of the animals to mechanical stimuli applied on glabrous plantar skin were tested. Vehicle-injected animals were treated identically and used as controls. Stimulation of the intact skin with mechanical forces of small intensities (10, 19, 36, 59 and 80 mN) did not elicit any paw withdrawal responses in the animals tested (Figure 3). In vehicle-treated control rats 1 hour after the surgery, stimulation of the glabrous skin with these small intensity filaments led to strong withdrawal reactions (Figure 3, triangle symbols). This increase of responsiveness reached statistical significance at many time intervals, depending on the stimulus intensity, and reached its maximum 2 - 6 h after the surgical procedure. A gradual return towards pre-incision values occurred over the next 6 days. The responsiveness of the contralateral intact hindpaws to these weak stimuli did not change from baseline after the intrathecal vehicle injection. These results suggest the presence of mechanical allodynia in the rat model of incisional pain used.

The application of VF filaments with stronger bending forces (144, 292 and 367 mN) evoked withdrawal responses in experimental animals also before the incision. Pre-incisional responsiveness did not differ between the hindpaws. As expected, the sensitivity to these stronger VF stimuli increased significantly after the surgery with all of these VF filaments. The increase of responsiveness peaked 2 - 6 h after the surgery and lasted for several days. Six days after the incision the responsiveness of the incised hindpaw was not different from that of the intact contralateral hindpaw, which remained unchanged during the experiment. These results suggest the presence of mechanical hyperalgesia induced by the plantar incision.

In another group of animals, 100 μM SB366791 solution was injected intrathecally 15 min prior to the incision (Figure 3, circle symbols). This almost did not change the increased responsiveness of the operated hindpaws to weak VF filaments compared to the vehicle-injected animals. Mechanical allodynia was significantly attenuated at only several time points by the intrathecal application of 100 μM TRPV1 receptor antagonist. The responsiveness to stimulation with VF filaments of more intense forces (144, 292 and 367 mN) was not affected significantly by the 100 μM SB366791 treatment. These results suggest that mechanical hyperalgesia was not attenuated by the intrathecal TRPV1 receptor antagonist treatment.

### The effect of intrathecal 10 μM SB366791 treatment on postincisional mechanical allodynia and hyperalgesia

**Figure 4:**
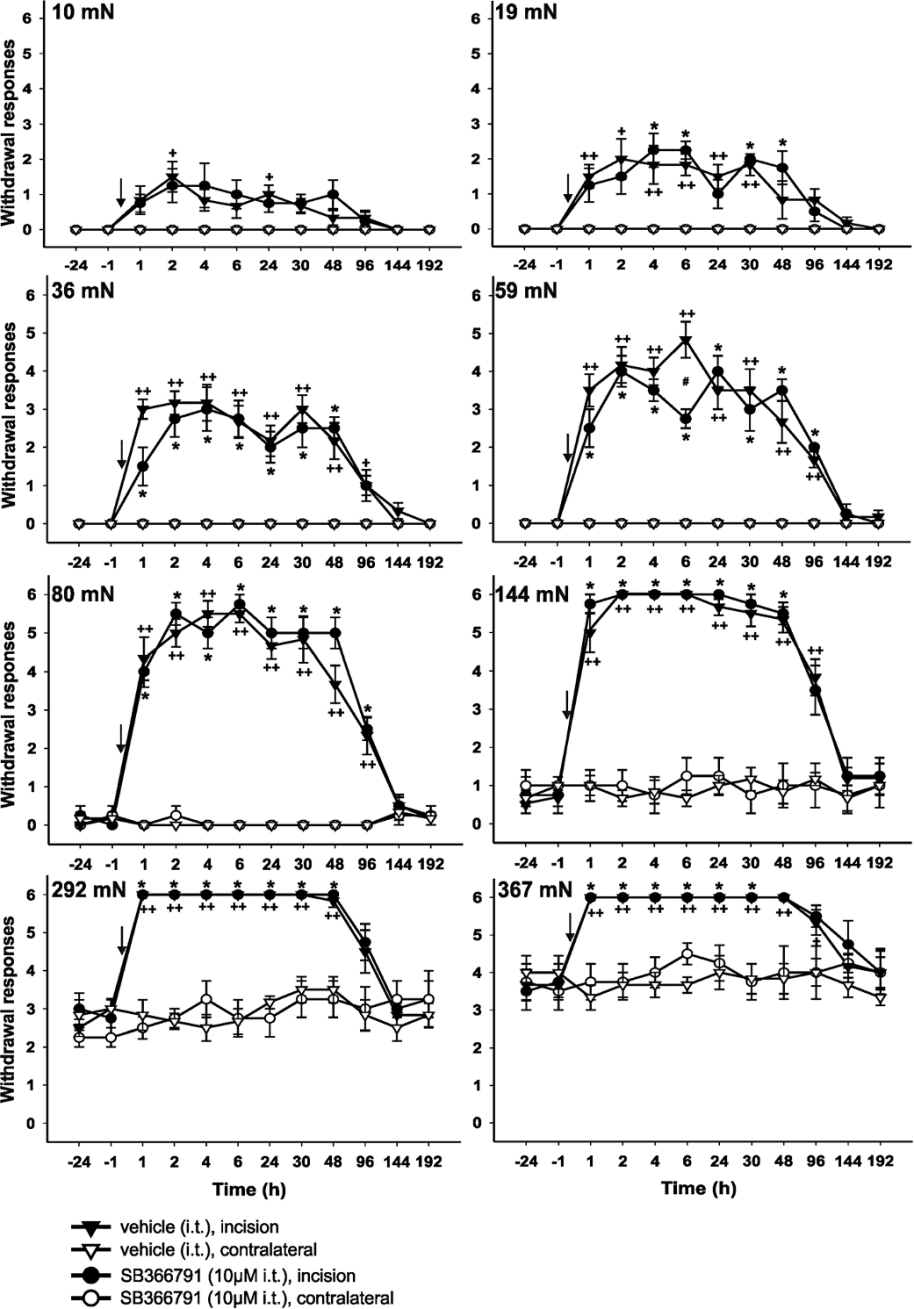
**The effect of intrathecal 10 μM SB366791 treatment on the responsiveness to mechanical stimuli after plantar incision.** Testing with a range of von Frey filaments after the intrathecal injection of vehicle (15 min before the surgery) indicated the development of postoperative mechanical allodynia (10, 19, 36, 59 and 80 mN) and hyperalgesia (144, 292, 367 mN) on the incised hind paw (n = 6). Intrathecal injection of 10 μM SB366791 (15 min before the surgery) (n = 4) showed only a modest antinociceptive effect on postoperative mechanical allodynia, while mechanical hyperalgesia remained unaffected. *P <0.05, SB366791 injection: incision versus contralateral side; ^+^P <0.05, ^++^P <0.01, vehicle injection: incision versus contralateral side; ^#^P <0.05 SB366791 versus vehicle injection on the incised hind paw. The time of drug administration is marked by arrow.

After the intrathecal application of 10 μM SB366791 solution (Figure 4, circle symbols) and the surgery, an increase of responsiveness of the operated hindpaws to weak VF filaments was observed, which was similar to the postoperative responsiveness of the control, vehicle-injected (Figure 4, triangle symbols) animals. The TRPV1 antagonist application reduced the responsiveness to VF filaments (59 mN) only moderately and transiently with statistical significance at a single testing time. The responsiveness to stimulation with VF filaments of more intense forces (144, 292 and 367 mN) and the development of postincisional mechanical hyperalgesia were not affected by intrathecal 10 μM SB366791 pretreatment when compared to the postoperative responsiveness of the incised hindpaws of vehicle-treated animals.

### Intradermal 100 μM SB366791 treatment attenuated postincisional mechanical allodynia

The role of peripheral TRPV1 receptors in the development of postincisional tactile hypersensitivity by the intradermal application of SB366791 30 min before and immediately after the surgical incision was tested. Vehicle-injected animals were treated identically and used as controls.

**Figure 5:**
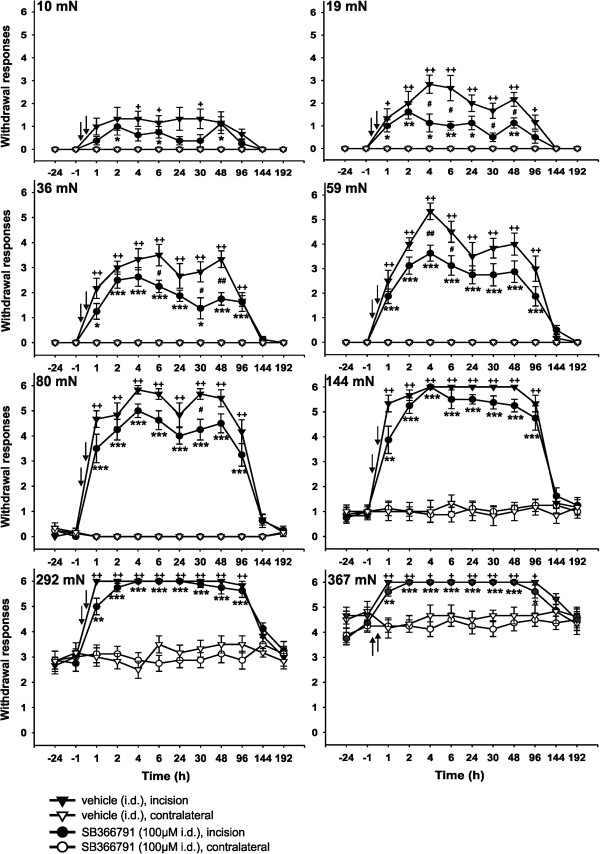
**Intradermal injection of TRPV1 receptor antagonist attenuated mechanical hypersensitivity induced by plantar incision.** A range of von Frey filaments applied to the incised skin after intradermal injection of vehicle (30 min before and immediately after the surgery) indicated the development of postoperative mechanical allodynia (10, 19, 36, 59 and 80 mN) and hyperalgesia (144, 292, 367 mN) while it did not affect the corresponding contralateral intact hindpaw (n = 6). Intradermal injection of 100 μM SB366791 (30 min before and immediately after the surgery) showed an antinociceptive effect of the TRPV1 receptor antagonist on postoperative mechanical allodynia, while mechanical hyperalgesia remained unaffected (n = 8). *P <0.05, **P <0.01, ***P <0.001 SB366791 injected incised skin versus contralateral; ^+^P <0.05, ^++^P <0.01, vehicle injected incised skin versus contralateral; ^#^P <0.05, ^##^P <0.01 SB366791 versus vehicle injected incised skin. The time of drug administration is marked by arrow.

Intradermal injection of the vehicle (Figure 5, triangle symbols) did not prevent increased responsiveness to weak VF filaments after the incision. In comparison, the responsiveness of the operated hindpaws after the intradermal injection of 100 μM SB366791 (Figure 5, circle symbols) solution was significantly attenuated after the incision. These results suggest reduced mechanical allodynia following the intradermal application of the TRPV1 receptor antagonist.

However, the responsiveness of the incised hindpaws to stimuli of more intense forces (144, 292 and 367 mN) was not affected significantly by the intradermal injection of 100 μM SB366791 compared to vehicle-pretreated incised hindpaws, suggesting no effect of locally administered TRPV1 receptor antagonist on mechanical hyperalgesia. The responsiveness to all mechanical stimuli of the contralateral intact hindpaws remained unchanged in both the control and experimental groups of animals.

### Application of the TRPV1 receptor antagonist intradermally or intrathecally did not affect mechanical sensitivity in naïve rats

To test the role of peripheral and central TRPV1 receptors in naïve rats, the basal reactivity to mechanical stimuli of 292 and 367 mN applied on glabrous plantar skin was examined under different conditions. The effect of 100 μM and 10 μM TRPV1 antagonist SB366791 applied intrathecally as well as the effect of 100 μM SB366791 applied intradermally on basic mechanical sensitivity were tested. Vehicle-injected animals were treated identically and used as controls.

Stimulation of the intact skin with mechanical forces of 292 and 367 mN evoked paw withdrawal responses in all the animals tested. This responsiveness did not differ between the hindpaws before and after either intrathecal or intradermal drug application. In the experimental groups of animals, either 100 μM or 10 μM SB366791 solution was injected intrathecally 15 min prior to the sham surgery or 100 μM SB366791 solution was injected intradermally 30 min before and immediately after sham surgery. In all these experimental groups of animals, there was no change of responsiveness of any of the intact hindpaws to these VF filaments (292 mN and 367 mN) observed when compared to the responsiveness of the vehicle-treated animals. Neither hypo- nor hypersensitivity to these mechanical stimuli was observed at any testing time caused by the intrathecal application of 100 μM or 10 μM or the intradermal injection of 100 μM TRPV1 receptor antagonist.

## Discussion

In the described behavioral experiments, we have investigated the contribution of spinal and peripheral TRPV1 receptors to the development of thermal and mechanical hypersensitivity in a rat model of postoperative pain. Plantar skin and underlying muscle incision rapidly induced a robust, long-lasting increase in sensitivity to thermal and mechanical stimuli, as was described previously [6, 38, 39]. The use of von Frey filaments of different strengths enabled us to distinguish between mechanical allodynia, using filaments that did not evoke responses before the incision (10 - 80 mN), and mechanical hyperalgesia, using filaments that did evoke responses before the incision (144 - 367 mN), similarly to our previous study [32].

Intrathecal pretreatment with 100 μM SB366791 dramatically attenuated the thermal hyperalgesia induced by plantar incision. Moreover, this i.t. TRPV1 receptor antagonist pretreatment induced transient hypoalgesia that was also apparent in the unoperated contralateral hind paw and in naïve animals. The analgesic effect of SB366791 was dependent on its concentration, as the effect of a 10 μM SB366791 i.t. injection on postincisional thermal hyperalgesia was only moderate. In comparison to i.t. pretreatment, intradermal injection of 100 μM SB366791 only moderately attenuated the thermal hyperalgesia induced by the plantar incision. These results suggest an important contribution of both the cutaneous and the spinal TRPV1 receptors in the development of postincisional thermal hypersensitivity, while intrathecal TRPV1 receptor antagonist pretreatment was more effective. We cannot exclude that this could be also due to incomplete inhibition of the affected TRPV1 receptors in the periphery after the intradermal application, including injured muscle nociceptors. In the present study with the TRPV1 antagonist, we have used identical methodological procedures to our previous study, where we used intradermal capsaicin treatment. This enabled us to compare these two local treatments with different mechanism of action. In this comparison, intradermal injection of capsaicin, presumably leading to epidermal nerves inactivation, had much more robust analgesic effect [32].

The importance of TRPV1 receptors for the development of thermal hyperalgesia after incision was shown previously using TRPV1 knockout mice [28, 29]. It was also suggested that the mechanism underlying the thermal hyperalgesia induced by incision may be largely mediated by the functional up-regulation of TRPV1 receptors in IB4-binding dorsal root ganglion (DRG) neurons with C-fibers [40]. The analgesic effect of systemically administered TRPV1 receptor antagonists A425619, ABT102, AMG0347, AMG9810, BCTC and SB705498, with different degrees of penetration in the central nervous system (CNS) ranging from very low to high, on thermal hyperalgesia induced by incision was demonstrated previously [41–44]. All TRPV1 receptor antagonists diminished incision-induced thermal hyperalgesia without its complete attenuation. The degree of CNS penetration of the antagonists did not significantly influence the analgesic effect in these studies. In our work we have demonstrated the almost complete alleviation of thermal hyperalgesia (with the exception of the 4^th^ postsurgical day) after a single intrathecal dose of TRPV1 antagonist administered before the surgery as a pretreatment. Based on a comparison with studies in which the TRPV1 antagonist was applied after the surgery [41–44], it seems that this could be an important factor in the efficacy of TRPV1 receptor antagonist treatment. Our results indicate that directly targeting spinal TRPV1 receptors may have an analgesic effect in cases of post-surgical pain. We expect that the major effect after the i.t. administration of SB366791 was mediated by TRPV1 receptors expressed on the presynaptic endings of the central branches of DRG neurons where the majority of TRPV1 receptors in the spinal cord are expressed [45, 46]. However, we cannot exclude possible diffusion of the drug along the dorsal roots into the spinal ganglia. Based on the TRPV1 receptors distribution and the time scale of the observed changes we would not expect that this diffusion played a significant role in our experiments. The hypoalgesic effect observed after the intrathecal application of TRPV1 antagonist in our experiments suggests the presence of TRPV1 receptor activation in the spinal cord under control conditions in naïve animals. Our and others’ earlier electrophysiological recordings did not suggest tonic activity of spinal TRPV1 receptors under control conditions [47, 48]. However, in a recent study by Park et al. [49], the authors demonstrated moderate tonic activity of spinal presynaptic TRPV1 receptors in control mice.

The effect of TRPV1 antagonist application on increased mechanical sensitivity was weak. Intrathecal and intradermal injections of SB366791 in our experiments showed only a limited analgesic effect on postincisional mechanical allodynia, without affecting hyperalgesia. The blockade of cutaneous TRPV1 receptors was more effective in attenuating mechanical hypersensitivity compared to spinal receptor inhibition. Both intrathecal and intradermal applications of SB366791 did not affect tactile sensations in naïve rats. Previous reports demonstrated a weak attenuation of plantar incision-induced mechanical hypersensitivity by oral administration of A425619 [42] and no effect of a single oral administration of AMG0347 [43] or ABT102, whereas repeated once daily dosing of ABT102 produced a significant reversal on the 8th day after surgery. The analgesic effect of ABT102 was not enhanced when used as a pretreatment [41]. However, in TRPV1 knockout mice the development of mechanical hypersensitivity induced by incision was preserved [28, 29]. These findings indicate that cutaneous and spinal TRPV1 receptors only partially contribute to the development and maintenance of mechanical allodynia but that other mechanisms, which involve activation of EP1 receptors [19], p38 MAPK [25] and BDNF upregulation [20] are also important in these processes.

In the plantar incision model, changes in the damaged peripheral tissues are the primary source of nociceptive activity, and some may also influence the activation of peripheral TRPV1 receptors. The surgery leads to a rapid local acidosis strongly correlating with the hypersensitive period [9], and an increase in NGF expression in the skin was shown to contribute to the paw guarding pain behavior and thermal hyperalgesia [10, 11, 50]. NGF leads to a robust increase of proton-evoked currents, substantially decreases the thermal threshold for TRPV1 receptor activation [51] and stimulates the insertion of TRPV1 receptors into the plasmatic membrane [52]. Other NGF family ligands including BDNF, neurotrophin-3 (NT-3) and neurotrophin-5 (NT-5) could also be significant players in postoperative pain [50]. The glial cell line-derived neurotrophic factor (GDNF) family ligands GDNF, artemin and neurturin enhanced capsaicin-stimulated release of CGRP and capsaicin-evoked Ca^2+^ transients in cultured DRG neurons [53] and combined hindpaw injection of artemin and NGF induced hyperalgesia lasting for 6 days [54]. Plantar incision increased GDNF in the muscle while artemin was increased in both skin and muscle [50]. Transgenic mice that overexpress artemin in the skin had increased levels of mRNA encoding TRPV1, GDNF family receptor α 3 (GFRα3) and NGF activated receptor TrkA in DRG neurons [55]. Interleukins (IL-1β, IL-6 and IL-10) were upregulated in skin and muscle after incision [50], while IL-6 can enhance the activity of the TRPV1 receptor [56]. Also, the activation of peripheral bradykinin receptors (B1, B2), P2X purinoceptors, cyclooxygenase (COX) and nitric oxide (NO) synthase [13, 57] was shown to have a role in incisional thermal hyperalgesia. Bradykinin, prostaglandins and ATP were found to sensitize the TRPV1 receptor to heat [58–60], while NO was shown to activate the TRPV1 receptor via S-nitrosylation of cysteine residues [61]. This substantial evidence indicates the possible sensitization/activation of peripheral TRPV1 receptors by many factors such as protons, neurotrophins (NGF, BDNF, NT-5, GDNF, artemin, neurturin), ATP, bradykinin, prostaglandins, NO and/or IL-6 after plantar incision, when a lowered threshold for TRPV1 receptor activation by temperature could have an important role.

The mechanisms underlying the possible sensitization and activation of spinal TRPV1 receptors [46] after plantar incision are more elusive, as changes in mediators and their receptors in the spinal cord have been much less investigated in this model. Upregulation of COX-1 and COX-2 in the lumbar spinal cord of the rat was induced by plantar incision [17, 18], and similarly a transient upregulation of COX-2 and NO synthase was found after surgery in sheep [62]. Surgical incision induced microglia and astrocyte activation and the segmental upregulation of interleukin-1beta (IL-1β) in the rat spinal cord [21], and BDNF upregulation in the spinal cord has also been shown [20]. It is not clear if the overexpression of artemin in the periphery, which upregulated TRPV1, GFRα3 and TrkA receptors in DRG neurons [55], could also induce an upregulation of these receptors in the central branches of primary afferents in the spinal cord. The GDNF family ligands GDNF, artemin and neurturin enhanced the capsaicin-stimulated release of CGRP in acute spinal cord slices [53]. These neurotrophic factors sensitize sensory neurons via the TRPV1 receptor [53, 54]. Spinal CCL2 contributes to the maintenance of postsurgical mechanical hypersensitivity [34], while inhibition of spinal TRPV1 receptors prevents the thermal hyperalgesia induced by CCL2 [63]. Upregulation of spinal TRPV1 receptors in the central terminals of DRG neurons and their increased sensitivity to endogenous agonist [48] could play an important role in the induction and maintenance of hypersensitivity after plantar incision.

In our experiments we have demonstrated the importance of spinal cord TRPV1 receptor activation for the development of thermal hyperalgesia after peripheral incision. Surgical incision induces numerous changes in both peripheral and spinal cord tissues, and many of these changes may also converge their effects through the TRPV1 receptor. A cocktail of neurotrophic factors, bradykinin, protons and other molecules are most likely crucial for increased TRPV1 receptor activation in the periphery, while the detailed mechanisms of spinal TRPV1 receptor activation after surgical incision remain to be identified.

## Methods

Adult male Wistar rats (200 – 250 g) were kept in plastic cages with soft bedding with access to food and water *ad libitum*, maintained on a 12 hr light, 12 hr dark cycle. In total, 56 animals were used in this study. The animals had been handled for 4 - 5 days prior to the experiment in order to familiarize them with the experimental environment and procedures. All experiments were approved by the local Institutional Animal Care and Use Committee and were consistent with the guidelines of the International Association for the Study of Pain, the National Institutes of Health Guide for the Care and Use of Laboratory Animals, the U.K. Animals (Scientific Procedures) Act, 1986 and associated guidelines, and the European Communities Council Directive of 24 November 1986 (86/609/EEC).

All efforts were made to minimize animal suffering, to reduce the number of animals used, and to utilize alternatives to *in vivo* techniques, if available.

### Behavioral test procedures

Responsiveness to mechanical stimulation was tested with von Frey (VF) filaments. Each VF monofilament was calibrated on a top-loading electronic balance and the force needed to bend the filament was measured. The calibration of the filaments was re-checked both before and at the end of each experiment to ensure that the stimulus intensity remained unchanged. Rats were placed on an elevated plastic mesh (0.5 × 0.5 cm perforations) under a nonbinding, clear plastic cage and were left to adapt to the testing environment for at least 15 min. VF filaments with bending forces of 10, 19, 36, 59, 80, 144, 292 and 367 mN were used to deliver punctuate mechanical stimuli of varying intensity to the plantar aspect of each hindpaw, from below the mesh floor. Each stimulus was applied six times, each poke spaced 2 s apart, and sequential monofilaments were applied in ascending order of stiffness. Care was taken to stimulate certain location on the plantar surface just next to the incision injury and the same area on the non-injured hindpaw. The number of withdrawal responses to the VF filament stimulation was recorded. Shifts in weight or voluntary movements associated with locomotion were not counted as a withdrawal response. Baseline responses were determined in all animals before any experimental procedure.

Responsiveness to thermal stimulation was tested with radiant heat applied to the plantar surface of each hindpaw. Rats were placed under a nonbinding, clear plastic cage on a clear 3 mm thick glass plate, elevated to allow maneuvering of a controlled, radiant heat source underneath. Each rat was left to adapt to the testing environment for at least 15 min prior to any stimulation. A focused light source with halogen bulb was used to deliver the heat stimuli (50 W, Dittel, Prague). The radiant heat was applied to the plantar surface of the hindpaw, in the area where the intradermal injection and the incision were applied and in the same area on the non-injured hindpaw. The hindpaw withdrawal latencies were measured with a digital timer. A 30 s cutoff time was imposed on the stimulus duration to prevent tissue damage. Withdrawal latencies were tested 3 times in each hindpaw with at least 5 min between the trials. Baseline withdrawal latencies were determined in all animals before any experimental procedure. The person performing the behavioral test was always blinded to the type of treatment.

### Intrathecal catheter implantation

Catheters were made of PE-5 tubing. One end of the PE-5 tube was connected to PE-10 tubing using epoxy-glue, and the tubing was filled with sterile physiological saline. For intrathecal catheter placement the animals were anesthetized with ketamine (100 mg/kg i.p., Narkamon, Zentiva) and xylazine (10 mg/kg i.m., Rometar, Zentiva). The surgery was performed in sterile manner. The back of the animal was clipped with an electric razor, a longitudinal incision through the skin and subcutaneous tissue above the spine was made and the upper lumbar vertebrae were exposed. The PE-5 end of the catheter was placed into the lumbar subarachnoid space (approximately 0.5 cm in length) and fixed to the spine with dental cement (Duracryl, Spofa). The wound was surgically closed in layers, and the PE-10 end of the catheter was exposed on the skin surface of the animal’s back and heat-coagulated. All the animals were tested before the control experiments during the behavioral acclimatization period for any signs of any neurological deficits due to the catheter implantation. None of the animals used in the experiments showed any neurological deficits. Animals were left to recover in their cages for at least 5 days. The position of the catheters was verified by a dye injection at the end of each experiment. The dosage of SB366791 for i.t. administration was determined according to our previous study [63].

### Local pretreatment of the plantar skin

The plantar skin of the experimental hindpaw was marked with a 1 cm long line in the middle of the plantar surface, starting at the proximal edge of the heel and extending towards the toes, with a permanent marker. This marked area served as the injection and/or incision site. Intradermal injections of SB366791 or vehicle, (0.1 ml) were delivered with a 27G needle on the plantar aspect of the hindpaw under short-lasting ether anesthesia. During the injection the needle penetrated the skin just at the distal end of the marked line. The injected volume spread sufficiently to raise a small blister in the skin, within which the incision was later made. SB366791 (Tocris) stock solution was dissolved in ethanol. The 100 μM application solution was prepared from the stock solution and 0.9% NaCl solution 1:9. SB366791 or control vehicle (0.9% NaCl with ethanol) was injected 30 minutes before and immediately after the incision. The dosage of SB366791 for i.d. injections was determined based on previous studies [64–67].

### Plantar incision model of postoperative pain

A model of surgical pain similar to that described previously [6, 32] was used. A longitudinal incision of the skin and underlying plantar muscle was made in a sterile manner under ether anesthesia. The 7 mm incision was made with a number 11 surgical blade, through the skin and fascia on the plantar aspect of the foot, starting approximately 3 mm from the proximal edge of the heel and extending towards the toes. The plantar muscle was incised longitudinally and the muscle origin and insertion remained intact. After hemostasis produced with gentle pressure, the skin was sutured with 2 stitches of non-absorbable monofil 4-0 on a DS19 needle. After surgery the animals were left to recover in their cages.

### Experimental groups

One group of animals was tested for baseline responses to mechanical and thermal stimuli and then either 100 μM SB366791 (n = 8) or vehicle without the TRPV1 antagonist (n = 6) was injected in one hindpaw. Thirty minutes later the plantar incision was made on the injected hindpaw. Immediately after suturing, another two intradermal injections of SB366791 were made with half of the volume being injected on each side of the incision. Care was taken that the liquid would not leak from the suture. The responses were then tested again 1, 2, 4, 6, 24, 30, 48, 96, 144 and 192 hours later. The second group of animals received the identical drug treatment (100 μM SB366791, n = 4 or vehicle, n = 6) while an interval of 30 min was between injections. No surgical incision of the hindpaw was made in these sham animals. They underwent the same ether anesthesia and behavioral testing procedure as was used in the experimental group where an incision was performed.

In the third group of animals the intrathecal catheters were implanted first. After 5 - 7 days of recovery, these animals were tested for baseline responses to mechanical and thermal stimuli. On the first day of the experiment, 20 μl of either SB366791 (100 μM, n = 6 or 10 μM, n = 4) or vehicle (n = 6) followed by 50 μl of physiological saline were applied into the catheter. Fifteen minutes later the plantar incision of the right hind paw was made, and the animals were further tested for responsiveness to mechanical and thermal stimuli according to the same testing protocol as described above. The fourth group of animals was also tested 5 - 7 days after intrathecal catheter placement. Following the intrathecal administration of either TRPV1 antagonist (20 μl of 100 μM SB366791, n = 6 or 10 μM SB366791, n = 4) or vehicle (n = 6) followed by 50 μl of physiological saline, there was no surgical incision of the hind paw made and the animals underwent identical ether anesthesia and behavioral testing as described above.

### Data analysis

The withdrawal responses evoked during the mechanical stimulation with VF filaments were evaluated as present (1) or absent (0) and a mean value from the 6 trials for each filament strength was calculated. The mean values from all the rats in the group were then averaged and means ± SEM were calculated. Paw withdrawal latencies (PWLs) evoked by heat stimuli were averaged from the 3 trials for each hindpaw, and means ± SEM were calculated for each experimental situation and time point. The data were examined using SigmaStat software, with Mann-Whitney rank test to analyse statistical differences at each different time point between the groups.
